# Exploring ChatGPT's potential for augmenting post-editing in machine translation across multiple domains: challenges and opportunities

**DOI:** 10.3389/frai.2025.1526293

**Published:** 2025-05-01

**Authors:** Jeehaan Algaraady, Mohammad Mahyoob

**Affiliations:** ^1^Department of Languages and Translation, Taiz University, Taiz, Yemen; ^2^Department of Languages and Translation, Taibah University, Madina, Saudi Arabia

**Keywords:** post-editing, machine translation, ChatGPT-4o, natural language processing, artificial intelligence, LLMS

## Abstract

**Introduction:**

Post-editing plays a crucial role in enhancing the quality of machine-generated translation (MGT) by correcting errors and ensuring cohesion and coherence. With advancements in artificial intelligence, Large Language Models (LLMs) like ChatGPT-4o offer promising capabilities for post-editing tasks. This study investigates the effectiveness of ChatGPT-4o as a natural language processing tool in post-editing Arabic translations across various domains, aiming to evaluate its performance in improving productivity, accuracy, consistency, and overall translation quality.

**Methods:**

The study involved a comparative analysis of Arabic translations generated by Google Translate. These texts, drawn from multiple domains, were post-edited by two professional human translators and ChatGPT-4o. Subsequently, three additional professional human post-editors evaluated both sets of post-edited outputs. To statistically assess the differences in quality between humans and ChatGPT-4o post-edits, a paired *t*-test was employed, focusing on metrics such as fluency, accuracy, coherence, and efficiency.

**Results:**

The findings indicated that human post-editors outperformed ChatGPT-4o in most quality metrics. However, ChatGPT-4o demonstrated superior efficiency, yielding a positive *t*-statistic of 8.00 and a *p*-value of 0.015, indicating a statistically significant difference. Regarding fluency, no significant difference was observed between the two methods (*t*-statistic = −3.5, *p*-value = 0.074), suggesting comparable performance in ensuring the natural flow of text.

**Discussion:**

ChatGPT-4o showed competitive performance in English-to-Arabic post-editing, particularly in producing fluent, coherent, and stylistically consistent text. Its conversational design enables efficient and consistent editing across various domains. Nonetheless, the model faced challenges in handling grammatical and syntactic nuances, domain-specific idioms, and complex terminology, especially in medical and sports contexts. Overall, the study highlights the potential of ChatGPT-4o as a supportive tool in translation post-editing workflows, complementing human translators by enhancing productivity and maintaining acceptable quality standards.

## Introduction

Machine translation (MT) has a significant role in facilitating communication and enhancing global interactions. This role has gained more attention in various contexts, driven by remarkable natural language processing technology advancements that enabled more efficient translation (Raj et al., 2023). However, MT outputs must be post-edited to ensure their desired quality and meet productivity standards. Translation post-editing (TPE) is a critical step in the translation process that involves reviewing and refining machine-translated content. Post-editing is not a recent trend, and it emerged in the earlier days of MT (Vieira, 2019). Recently, post-editing MT gained considerable interest as a service and research topic due to the advancements in translation technology. Post-editing implies correcting grammatical errors in vocabulary, improving sentence structure, adjusting tone and style, ensuring cultural appropriateness, and refining the translation to align with the intended purpose and audience (Daems et al., 2013; Vardaro et al., 2019). Moreover, it allows for a more customized and tailored approach to translation, as post-editors can adapt the output to meet specific clients. According to Allen (2001), post-editing is correcting and refining the machine-generated translation (MGT) after translation from a source to a target language.

There are several types of post-editing, each catering to the number of corrections, efforts, and objectives required to achieve the desired translation. An early study on post-editing typology by Laurian (1984) proposed two types of post-editing: rapid post-editing and conventional post-editing. The former involves correcting the translated texts without paying attention to the translation style, while the latter implies deep correction to produce a human-like translation.

Allen (2003) suggests two types of post-editing: minimal and complete PEs. Minimal PE is for quick review, focusing mainly on critical errors and ensuring essential language accuracy, controlled by limited time and budget. However, complete PE aims to perform deep corrections closely resembling human translation standards.

van Egdom and Pluymaekers (2019) and Vieira (2017) established four levels of post-editing: “minimal,” “light,” “moderate,” and “full,” precisely. For post-editing quality guidelines, the Translation Automation User Society (TAUS, 2010) differentiates between two standards of expected target-text quality: “good enough” quality and quality “similar or equal to human translation.” Indeed, these criteria almost correspond to “light” and “full” post-editing, respectively (Massardo et al., 2016). The TAUS guidelines stress that the level of post-editing depends on the deliberate purpose of the text and the quality of the raw MT output, making the target quality a more consistent factor for post-editing guidelines. Post-editors have no strict instructions about the issues they need to focus on. These instructions differ depending on whether they aim for “good enough” or “human translation quality.” When machine translation (MT) errors impact meaning, for “good enough” quality, the focus is on semantics and comprehensibility, with less consideration given to syntactic or grammar. Conversely, post-editors should address style, syntax, grammar, and formatting issues when focusing on human translation quality. Additionally, they should handle terms that need to remain in the original language but may have been translated by the MT system.

In MT, post-editing has two paradigms, including static and interactive. In the former, the machine generates translation in the first step and then edits it in the second. The latter implies real-time collaboration between translators and MT systems (Vieira, 2019). In terms of these two paradigms, there are different findings; for example, Langlais and Lapalme (2002), in their TransType tool evaluation, evoked that interactive post-editing could lead to reduced productivity by up to 35% compared to static editing. Koehn et al. (2015) stated that interactive models with online learning seemed to require less technical effort, with post-editors becoming faster over time. However, it has also been proven that interactive post-editing may not notably affect target-text quality and could even result in errors (Underwood et al., 2014). Compared to static post-editing, interactive post-editing may take longer but result in higher-quality products (Green et al., 2014).

With the advent of advanced Neural Network systems, the generated translation becomes more accurate and naturally sounding (Qin, 2022). However, these translations still have inaccuracies, errors, and inappropriate phrasing. It is a vital step that bridges the gap between automated generated translation and human editors and linguistic expertise to enhance translation fluency, coherence, and linguistic appropriateness.

The collaborative interaction between artificial intelligence and human intervention offers a cost-effective and efficient approach to high-quality translation services in various domains where translation quality is critical, especially for legal, medical, and technical content. With the proliferation of these technologies, research on large language models (LLMs) and linguistic analysis, particularly in fields such as second language acquisition (Albuhairy and Algaraady, 2025), learner error analysis (Al-Garaady and Mahyoob, 2023), natural language processing (Mahyoob and Al-Garaady, 2018; Mahyoob, 2020), and academic writing development (Mahyoob et al., 2023), has become increasingly critical.

Though human post-editors of MGTs show high-quality products, their work is time-consuming, and they challenge both balanced speed and quality. This research investigates how ChatGPT-4o, an advanced language generation model, can enhance translation post-editing productivity, efficiency, and quality across various domains and how human editors benefit from ChatGPT-4o in their TPE tasks.

### Research question

This work attempts to answer the following research questions as a starting point for exploring the role of ChatGPT-4o in various aspects of post-editing machine-generated translations.

1. Can ChatGPT-4o integration maintain human translators' productivity, consistency, and efficiency instead of a human editor during post-editing?
2. To what extent can ChatGPT-4o improve the overall quality of MGT through post-editing?
3. How does ChatGPT-4o's performance in post-editing compare to traditional post-editing methods?
4. What challenges and limitations are encountered when using ChatGPT-4o for post-editing in certain domains? Moreover, to what extent can these challenges be alleviated?
5. How much does using task-specific prompts improve ChatGPT-4o performance in PE?

## Literature review

MTPE is the process of reviewing and correcting errors in machine-generated translations. This section provides an overview of the literature on translation post-editing and integrating language models like ChatGPT-4o in translation workflows. It discusses the challenges faced in translation post-editing, advancements in machine translation PE technologies, and the role of artificial intelligence in improving translation PE quality.

Screen (2019) compared post-edited translations with translations created from scratch in the Welsh text. He said post-translation editing was not found to improve. The two types of products are mainly similar in terms of comprehension and readability, which supports the use of MT in professional settings.

A study conducted with software instructions translated from English to Brazilian Portuguese found that even minimal post-editing significantly increased the usability of MT-based texts. The improvements were measured using eye-tracking metrics and self-reported satisfaction, highlighting the value of post-editing in enhancing text comprehensibility and accuracy (Castilho et al., 2014).

Koneru et al. (2023) made an Initial adjustment for direct translation. Therefore, researchers propose to use LLM as an automatic post editor (APE) instead. With Low-Rank-Adapter fine-tuning, they refined sentence- and document-level indicators. The ContraPro test achieved an accuracy of 89% in Anglo-German translations. In addition, including human corrections in document-level translations reduced the need for corrections in translation. Raunak et al. (2023) used GPT-4 for automatic post-editing in language pairs. It was found that there was an improvement in the accuracy and reliability of the WMT-22 English-Chinese, English-German, Chinese-English, and German-English tasks. However, sometimes GPT-4 might cause incorrect edits that demand caution in utilization. Chen et al. (2023) recommend improving iterative translation using large-scale language models for advanced translation and post-editing, especially for complex structures. However, this method showed limited scalability and computational challenges. Moreover, the model relies heavily on pre-trained models.

IntelliCAT, introduced by (Lee et al., 2021), is an interactive translation interface designed to improve post-machine translation editing. It uses sentence-level and word-level quality estimation (QE) to predict sentence quality and identify errors for improvement. The translation recommendation model includes word and phrase alternatives, while word alignments preserve the original document format. Experiments show that these features advance translation quality. User studies confirm that post-editing is 52.9% faster than translation from scratch. Turchi et al. (2017) explored machine translation (MT) improvements using human post-editing within a Neural Machine Translation (NMT) framework, highlighting the benefits of batch method customization. Continuously, It enables real-time optimization of new users and domains at low computational cost. Various online learning strategies are tested to refine existing models based on input data and after modification. Evaluating two language pairs showed a significant improvement over the static model.

## Data collection and methodology

### Data collection

To conduct our exploration, this research utilized translation data comprising source texts (English) and their corresponding Arabic MGTs produced by a neural network-based machine translator (Google Translator). This dataset spans different domains to simulate real-world translation scenarios, including sports, medical, business, idioms, and literary texts, to ensure a comprehensive assessment of ChatGPT-4o's potential across various domains. As detailed in Table 1, the source texts were collected from several online platforms such as UN news^1^, Newatlas^2^, Saudigazette^3^, and American literature^4^, comprising 6,203 English words (ws). Their Arabic translations produced by Google Translate [GT (A)] amount to 5,582 ws, while the human post-editing version [H-PE(A)] includes 5,393 ws, and the ChatGPT4o post-editing version [C- PE(A)] contains 5,451 ws.

**Table 1:** Statistical description of the dataset.

**Texts**	**Sports**	**Business**	**Medical**	**Literary**	**Total**
Source (E)	1,580 ws	1,498 ws	1,539 ws	1,586 ws	6,203 ws
GT (A)	1,357 ws	1,283 ws	1,298 ws	1,564 ws	5,582 ws
H-PE (A)	1,332 ws	1,261 ws	1,258 ws	1,542 ws	5,393 ws
C- PE (A)	1,351 ws	1,268 ws	1,273 ws	1,559 ws	5,451 ws

### Experiment/method

In this experiment, first, the collected texts undergo initial translation from English into Arabic using a neural network-based machine translator (Google translator) to establish a baseline for comparison. Second, the generated translations are post-edited in two modes, first by two professional human translators and then using ChatGPT-4o as a post-editing tool. ChatGPT-4o is requested to improve and revise the MGT to explore and assess the extent of ChatGPT-4o's capabilities in performing or enhancing post-editing machine-translated content. The two human translators were given different sets of data to post-edit to boost the diversity of post-edited translations and interpretations that reflect the Arabic richness and capture a broader range of editorial perspectives.

Third, a panel of three human editors (HEs) manually validated and evaluated the improvements and suggestions provided by human translators and ChatGPT-4o. Fourth, we compare the quality of the post-edited content by human translators and the quality of the post-edited content by ChatGPT-4o based on a set of evaluation metrics using *T*-test statistics. In addition, we compare the performance of ChatGPT-4o across different domains to assess its domain adaptation capabilities. Indeed, knowing ChatGPT-4o's ability to provide post-editing for machine translation would help make a clear decision to incorporate ChatGPT-4o's post-editing service for various stakeholders who benefit from post-editing translation.

## Data analysis and evaluation

### Evaluation measures for ChatGPT-4o and human post-editing of MGT across multiple domains

In this section, we analyze the impact of ChatGPT-4o on machine translation post-editing (MTPE). Based on this analysis, we attempt to identify patterns, challenges, and areas for improvement. We comprehensively compare the different post-editing modes (professional translator's post-editing and ChatGPT-4o post-editing) in terms of several key evaluation measures, including fluency, accuracy, efficiency, terminology, consistency, coherence, grammar, culture, and appropriateness. Generally, these criteria and standards are used to evaluate and improve the quality of translation as a machine product. Our analysis offers insights into ChatGPT-4o's ability to complement human expertise in post-editing, highlighting its strengths and limitations in enhancing the quality and efficiency of translation workflows.

After it is edited from a machine translation (MT) output, a text's linguistic smoothness and naturalness improve. These metrics focus on readability, grammar, syntax, and flow. As illustrated in Table 2, in terms of fluency (concentrate on readability, grammar, syntax, and flow), in the sentence extracted from a business text, the MGT version (a Google translate's generated translation) looks straight up, simple, and lacks fluency but still work as evaluated by HE. However, to some extent, when prompting ChatGPT-4o to evaluate the machine-generated translation MGT sentence structures for the source version (S), the ChatGPT-4oE version follows the natural flow of language compared to MGT, though it is not perfect like that in the HE version. ChatGPT-4oE provides a contextual version due to its conversational nature, enhancing the performance of translation studies. For accuracy, the ChatGPT-4o post-edited version shows proper punctuation usage. There are no spelling errors or typos, but there are slight errors in the translation grammar, including functional words usage such as articles as in ChatGPT-4oE phrase/“الصندوق النقد الدولي,” “IMF”/, where it adds the article/“the,”ال“ /in the word “الصندوق” inappropriately though it is correct in MGT version. However, the post-edited version by humans looks more cohesive as it maintains the coherence between sentences and paragraphs compared to the original version translated by Google Translate and the post-edited version by ChatGPT-4o.

**Table 2:** A sample of MGT, ChatGPT-4o's post-editing of MGT, and human's post-editing of MGT for business text.

**S**	**The latest estimate is lower than the 3.1 percent GDP growth projected by the IMF in May**
MGT	في المائة الذي توقعھ صندوق النقد الدولي في مایو. 3.1 التقدیر الأخیر أقل من نمو الناتج المحلي الإجمالي بنسبة
ChatGPT-4oE	.ویعتبر ھذا التقدیر الأخیر أقل من التوقعات السابقة بنسبة . ٣٪ لنمو الناتج المحلي الإجمالي التي أعلنھا الصندوق النقد الدولي في شھر مایو
HE	.٪ویعتبر ھذا التقدیر الأخیر لنمو الناتج المحلي الإجمالي اقل مما توقعھ صندوق النقد الدولي سابقا في مایو بنسبة ٣.

Table 3 shows the output of the ChatGPT-4o post-editing of the literary text containing an idiomatic expression. It provides effective post-editing, showing substantial grammar, sentence structure, and readability improvements. However, it failed to maintain the idiomatic meaning for an Arabic audience. It provides accurate, unique literary phrases specific to the Arabic language that often carry cultural, historical, and contextual significance. As shown in the example below, both MGT and ChatGPT-4oE provide Arabic literary translation الطيور تتجمع'' معًا for the idiom “Birds Of A Feather Flock Together.” that is postedited by the human translator as/“Birds Of A Feather Flock Together, “الطيور على اشكالها تقع”/ and reflects a common challenge in AI's handling of nuanced and culturally specific elements and the need for AI to evolve beyond essential linguistic translations to encompass cultural interpretations, demanding more sophisticated training datasets and model enhancements.

**Table 3:** A sample of MGT, ChatGPT-4o's post-editing of MGT, and human's post-editing of MGT for idioms.

**S**	**The shared interests provide a strong foundation for friendship, as friends engage in meaningful conversations and activities they enjoy, as said in “Birds Of A Feather Flock Together”**
MGT	تشكل الاھتمامات المشتركة أساسًا قویًا للصداقة، حیث یشارك الأصدقاء الذین ینسجمون مع بعضھم البعض في محادثات وأنشطة ھادفة یستمتعون بھا. وكما یقولون: "الطیور تتجمع معًا.
ChatGPT-4o E	“.الاھتمامات المشتركة تشكل أساساً قویاً للصداقة، حیث یشارك الأصدقاء في محادثات ذات مغزى وأنشطة ھادفة یستمتعون بھا معًا، كما یقال: 'الطیور تتجمع معًا.
HE	من منطلق المثل القائل: الطیورعلى اشكالھا تقع, تشكل الاھتمامات المشتركة أساسًا قویًا للصداقة، حیث یستمتع الأصدقاء المنسجمون مع بعضھم بالمشاركة في محادثات وأنشطة ھادف”’.

As illustrated in Table 4, in the case of the medical terms, ChatGPT-4o failed to post-edit and provide the accurate Arabic equivalents for some medical terms; for example, ChatGPT-4oE provided literary translation for the phrase “healthcare errors” as “أخطاء الرعاية الصحية” of which the correct Arabic equivalent is “خطاء طبية.” In some cases, ChatGPT-4oE failed to provide any translation for these terms, such as the word “astigmatism.”. Instead, it gives transliteration for the term as “الاستجماتيزم.” while the correct Arabic translated term is “ الابؤرية” as edited by HE. In addition, there is a linguistic agreement error as seen in the anaphor (possessive pronoun “their”) in the phrase/“their health,” “صحتهم”/ which should be/“his health,” “صحته”/ since this phrase refers to the singular antecedent/“a person,” “الشخص”/. However, the anaphor generated by MT agreed with its antecedent. Compared to human editors, ChatGPT-4o failed to ensure and improve consistency in terminology and medical terms throughout the text.

**Table 4:** A sample of medical MGT, ChatGPT-4o's post-editing of MGT, and human's post-editing of MGT medical texts.

**S**	**If a person with astigmatism experiences a healthcare error, it may affect their overall wellbeing and exacerbate existing vision issues**
MGT	إذا تعرض الشخص المصاب بالاستجماتیزم لخطأ في الرعایة الصحیة، فقد یؤثر ذلك على صحتھ العامة ویؤدي إلى تفاقم مشكلات الرؤیة الحالیة.
ChatGPT-4oE	إذا تعرض الشخص المصاب بالاستجماتیزم لخطأ في الرعایة الصحیة، فقد یؤثر ذلك على صحتھم العامة وقد یزید من تفاقم مشكلات الرؤیة الحالیة.
HE	إذا تعرض الشخص المصاب بالابؤریة لخطأ طبي، فقد یؤثر ذلك على صحتھ العامة و یفاقم مشكلات الرؤیة الحالیة.

ChatGPT-4o struggles to produce an efficient translation in the case of literary texts, as seen in Table 5 below. There is a grammatical error where the singular noun “a secret” in the phrase “a secret has been …” is translated inappropriately to plural noun /“secrets,” “اسرار”/which should be translated to the Arabic singular noun “سر.” Also, the syntactic structures look inferior compared to MGT and HE versions. ChatGPT-4oE, in the case of literary texts, shows significant issues in using correct and consistent terms and looks poor in its language smoothness and naturalness, cohesion, grammar, cultural aspects, and terminology handling.

**Table 5:** A sample of MGT, ChatGPT-4o's post-editing of MGT, and human's post-editing of MGT for a literary text.

**S**	**I had an elderly neighbor whose conversations were mind-blowing. He told me one day when his gathering was devoid of people, and no one was sitting with us: “Oh my son, a secret has been stirring in my heart for forty years regarding what has been discussed during this lengthy period**
MGT	كان لدي جار مسن كانت محادثاتھ مذھلة. قال لي ذات یوم وقد خلا نادیھ من الناس ولم یكن بیننا من یجلس: یا بني إن سر ما ذكر في ھذه المدة الطویلة ما زال یتحرك في قلبي منذ أربعین سنة.
ChatGPT-4oE	1. كان لدي جار كبیر في السن كانت محادثاتھ مذھلة. قال لي یوماً وكان نادیھ خالیاً من الناس ولم یكن ھناك أحد یجلس بیننا: یا إبني، كانت أسرار ما تم ذكره خلال ھذه الفترة الطویلة تحرك في قلبي لمدة أربعین عامًا.
HE	كان لي جار من الكھول، یخلب بسماع أحادیثھ العقول، فقال لي ذات یوم — وقد خلا نادیھ من الحضور، ولم یكن معنا فیھ أحد من الجلَّاس: یا بُنيَّ، إنھ یختلج في صدري منذ أربعین سنة سرٌّ ما جرى ذكره في ھذه المُدَّة الطویلة على الألسنة.

Table 6 shows that ChatGPT-4o failed to appropriately edit the phrase (' in building bridges, “في بناء الجسور”) and provide the same MGT version (literal translation for this phrase). However, the HE version/“in building bridges,” “في بناء جسورالتواصل”/demonstrates a deeper and more accurate understanding and use of consistent terms. All these emphasize using ChatGPT-4o with caution in the translation industry because the HE edition emphasizes promoting proper contact and understanding between people, which is often implied when discussing “Building Bridges.” This version not only maintains the source phrase's true meaning but also enriches the meaning by adding a more nuanced layer of meaning that is more appropriate and resonant for the reader. In the case of the phrase/“whatever you feel in the Games,” “كل ما يجول بخاطرك حول الألعاب الرياضية”/, both MGT and ChatGPT-4o provide unnatural and inconsistent translation version/ما تشعر به في الألعاب” “كل ما تشعر به في الألعاب,”/compared to that provided by HE version.

**Table 6:** Sample of MGT, ChatGPT-4o post-editing of MGT, and human post-editing of MGT for sports text.

**S**	**Tsuyoshi Kitazawa, a former member of Japan's national football team, stressed the role of sport in building bridges: “whatever you feel in the Games is made possible because the world is playing as one team,” he said**
MGT	“وشدد تسویوشي كیتازاوا، العضو السابق في المنتخب الوطني الیاباني لكرة القدم، على دور الریاضة في بناء الجسور: "كل ما تشعر بھ في الألعاب أصبح ممكنا لأن العالم یلعب كفریق واحد.
ChatGPT-4oE	،“تسویوشي كیتازاوا، عضو سابق في منتخب الیابان الوطني لكرة القدم، أكد دور الریاضة في بناء الجسور: "ما تشعر بھ في الألعاب یصبح ممكنًا لأن العالم یلعب كفریق واحد
HE	واكد العضو السابق في المنتخب الوطني الیاباني لكرة القدم، تسویوشي كیتازاوا، على دور الریاضة في بناء جسور التواصل قائلا: " كل ما یجول بخاطرك حول الألعاب الریاضیة أصبح ممكنا لأن العالم أصبح یلعب كفریق واحد".

This demonstrates that ChatGPT-4o fails to communicate the deeper intent to the audience effectively. ChatGPT-4o provides accurate numbers, information, and proper names. However, concerns include sentence structure using compound words, function words, and word ordering, as seen in Table 6. All of this highlights the careful use of ChatGPT-4o in the translation industry.

### Prompt engineering for enhancing ChatGPT-4o outcomes

Mostly, it is noticed that the performance of ChatGPT-4o becomes more meaningful and more profound when we specify the needs and provide context, background, and a comprehensive input “prompt.” For example, giving these details, “post-edit the Arabic generated translation below from the linguistic perspective, take the role of a professional grammar corrector, identify business terms, avoid changing meaning as much as possible” to the prompt enhances the tool's outputs. This can be seen in the improvements in ChatGPT-4oE 2 in Table 7, where the article “the, ال” is appropriately used compared to that in the ChatGPT-4oE 1 in the phrase/اصندوق النقد “الدولي,” “IMF”/.

**Table 7:** ChatGPT-4o post-editing with business texts after prompt engineering.

**S**	**The latest estimate is lower than the 3.1 percent GDP growth projected by the IMF in May**
MGT	التقدیر الأخیر أقل من نمو الناتج المحلي الإجمالي بنسبة 3.1 في المائة الذي توقعھ صندوق النقد الدولي في مایو.
ChatGPT-4oE	1. ویعتبر ھذا التقدیر الأخیر أقل من التوقعات السابقة بنسبة . ٣٪ لنمو الناتج المحلي الإجمالي التي أعلنھا الصندوق النقد الدولي في شھر مایو. 2. من قبل صندوق النقد الدولي في شھر مایو % 3.1 و یعتبرالتقدیر الأخیر أقل من معدل نمو الناتج المحلي الإجمالي المتوقع عند.
HE	.٪ویعتبر ھذا التقدیر الأخیر لنمو الناتج المحلي الإجمالي اقل مما توقعھ صندوق النقد الدولي سابقا في مایو بنسبة . ٣.

When we give these details “post-edit the Arabic generated-translation below from the linguistic perspective, take the role of a professional grammar corrector, identify idiomatic phrases, avoid changing meaning as much as possible” to the prompt of ChatGPT-4o in the literary texts, ChatGPT4o corrects its translation and post-editing. The yield results were more natural and accurate, as seen in ChatGPT-4o E 2 in Table 8, which shows improvement in the sentence flow compared to ChatGPT-4oE 1 due to some grammatical and stylistic adjustments. For example, the Arabic equivalent of the word “elderly” looks more fluent in the ChatGPT-4o E 2 version as “مسن” compared to that in the ChatGPT-4o E 1 “كبير في السن”. Also, the grammatical mistake in the ChatGPT-4o E 1 version is spotted in the ChatGPT-4o E 2 version, as the word “secret” is translated to a singular noun “سر” instead of plural noun “اسرار” like that in ChatGPT-4o E 1.

**Table 8:** ChatGPT-4o outcomes in literary texts after prompt engineering.

**S**	**I had an elderly neighbor whose conversations were mind-blowing. He told me one day when his gathering was devoid of people, and no one was sitting with us: “Oh my son, a secret has been stirring in my heart for forty years regarding what has been discussed during this lengthy period**
MGT	كان لدي جار مسن كانت محادثاتھ مذھلة. قال لي ذات یوم وقد خلا نادیھ من الناس ولم یكن بیننا من یجلس: یا بني إن سر ما ذكر في ھذه المدة الطویلة ما زال یتحرك في قلبي منذ أربعین سنة.
ChatGPT-4oE	1. كان لدي جار كبیر في السن كانت محادثاتھ مذھلة. قال لي یوماً وكان نادیھ خالیاً من الناس ولم یكن ھناك أحد یجلس بیننا: یا إبني، كانت أسرار ما تم ذكره خلال ھذه الفترة الطویلة تحرك في قلبي لمدة أربعین عامًا. 2. كان لدي جار مسن، محادثاتھ كانت استثنائیة. في یوم ما كان نادیھ خالیاً من الناس و لم یكن ھناك أحد آخرمعنا خلال تجمعنا، قال لي: 'یا بني، ھناك سر یدور في قلبي منذ أربعین عامًا بشأن المناقشات التي جرت خلال ھذه الفترة الطویلة.
HE	كان لي جار من الكھول، یخلب بسماع أحادیثھ العقول، فقال لي ذات یوم — وقد خلا نادیھ من الناس، ولم یكن معنا فیھ أحد من الجلَّاس: یا بُنيَّ، إنھ یختلج في صدري منذ أربعین سنة سرٌّ ما جرى ذكره في ھذه المُدَّة الطویلة على الألسنة

ChatGPT-4oE 1, in Table 9, displays the result of ChatGPT-4o outcomes when the prompt is “post-edit.” At the same time, ChatGPT-4oE 2 shows the ChatGPT-4o outcomes with a comprehensive prompt, “post-edit the Arabic generated translation below from the linguistic perspective, take the role of a professional grammar corrector, identify medical terms, avoid changing meaning as much as possible.” As seen in ChatGPT-4oE 2, the tool still shows a deficiency in providing the correct Arabic medical translated terms such as “الابؤرية” and “خطأ طبي” for the English medical terms “astigmatism” and “healthcare,” even though the tool is provided with a comprehensive prompt. The output in ChatGPT-4oE 2 looks identical to that provided without prompt engineering except for the omission of the article “the, ال” in words “person, شخص” and “affected, مصاب”. We notice grammatical and stylistic improvements in the ChatGPT-4oE 2 version compared to the ChatGPT-4oE 1 version, for example, the linguistic agreement error in the anaphora (possessive pronoun 'their') in the phrase/“their health,” “صحتهم”/is correctly translated to/“his health,” “صحته”/.

**Table 9:** ChatGPT-4o post-editing in medical after prompt engineering.

**S**	**If a person with astigmatism experiences a healthcare error, it may affect their overall wellbeing and exacerbate existing vision issues**
MGT	إذا تعرض الشخص المصاب بالاستجماتیزم لخطأ في الرعایة الصحیة، فقد یؤثر ذلك على صحتھ العامة ویؤدي إلى تفاقم مشكلات الرؤیة الحالیة.
ChatGPT-4oE	1. إذا تعرض الشخص المصاب بالاستجماتیزم لخطأ في الرعایة الصحیة، فقد یؤثر ذلك على صحتھم العامة وقد یزید من تفاقم مشكلات الرؤیة الحالیة. 2. اذا تعرض شخص مصاب بالاستجماتیزم لخطأ في الرعایة الصحیة, فقد یؤثر ذلك على صحتھ العامة وقد یؤدي الى تفاقم مشكلات الرؤیة الحالیة.
HE	إذا تعرض الشخص المصاب بالابؤریة لخطأ طبي، فقد یؤثر ذلك على صحتھ العامة و یفاقم مشكلات الرؤیة الحالیة.

In Table 10, the ChatGPT-4o E 2 version shows an enhanced, fluent, and natural post-editing that highlights the role of prompt engineering in raising the tool's advanced linguistic capabilities. This version shows an accurate idiomatic expression, particularly after adding a perspective and a contextual background to our prompt. Interestingly, ChatGPT-4o delivers a precise and culturally appropriate Arabic translation, “الطيور على أشكالها تقع” for the English idiom “Birds Of A Feather Flock Together”. However, the tool failed earlier in providing the appropriate Arabic equivalent idiomatic expression, as shown in ChatGPT-4o E 1.

**Table 10:** ChatGPT-4o post-editing with idioms after prompt engineering.

**S**	**The shared interests provide a strong foundation for friendship, as friends engage in meaningful conversations and activities they enjoy, as said in “Birds Of A Feather Flock Together”**
MGT	.تشكل الاھتمامات المشتركة أساسًا قویًا للصداقة، حیث یشارك الأصدقاء الذین ینسجمون مع بعضھم البعض في محادثات وأنشطة ھادفة یستمتعون بھا. وكما یقولون: "الطیور تتجمع معًا”
ChatGPT-4o E	1. “الاھتمامات المشتركة تشكل أساساً قویاً للصداقة، حیث یشارك الأصدقاء في محادثات ذات مغزى وأنشطة ھادفة یستمتعون بھا معًا، كما یقال: 'الطیور تتجمع معًا. 2. تشكل الاھتمامات المشتركة أساسًا قویًا للصداقة، حیث یشارك الأصدقاء الذین یتألفون في محادثات وأنشطة ھادفة یستمتعون بھا. وكما یقول المثل: "الطیور على أشكالھا تقع.”
HE	من منطلق المثل القائل: الطیورعلى اشكالھا تقع, تشكل الاھتمامات المشتركة أساسًا قویًا للصداقة، حیث یستمتع الأصدقاء المنسجمون مع بعضھم بالمشاركة في محادثات وأنشطة ھادفة.’.”

In Table 11, the ChatGPT-4oE 2 version resulted after providing the tool this enhanced prompt, “post-edit the Arabic generated translation below from the linguistic perspective, take the role of a professional grammar corrector, identify sport terms, avoid changing meaning as much as possible”. However, the structure of this version looks better; like ChatGPT-4oE 1 version, it failed to provide a suitable translation for the phrases, /“in building bridges,” “في بناء جسورالتواصل ”/ and / “whatever you feel in the Games,” كل ما يجول بخاطرك حول الألعاب الرياضية/ that highlights the limited role of ChatGPT4o in providing satisfied translation in specific sport-terms as some expressions require deep understanding.

**Table 11:** ChatGPT-4o post-editing with idioms after prompt engineering.

**S**	**Tsuyoshi Kitazawa, a former member of Japan's national football team, stressed the role of sport in building bridges: “whatever you feel in the Games is made possible because the world is playing as one team,” he said**
MGT	وشدد تسویوشي كیتازاوا، العضو السابق في المنتخب الوطني الیاباني لكرة القدم، على دور الریاضة في بناء الجسور: "كل ما تشعر بھ في الألعاب أصبح ممكنا لأن العالم یلعب كفریق واحد”.
ChatGPT-4oE	1. “تسویوشي كیتازاوا، عضو سابق في منتخب الیابان الوطني لكرة القدم، أكد دور الریاضة في بناء الجسور: "ما تشعر بھ في الألعاب یصبح ممكنًا لأن العالم یلعب كفریق واحد” 2. “وشدد تسویوشي كیتازاوا، اللاعب السابق في المنتخب الوطني الیاباني لكرة القدم، على أھمیة دور الریاضة في بناء الجسور قائلاً: "كل ما تشعر بھ خلال الألعاب أصبح ممكناً لأن العالم یلعب كفریق واحد”.
HE	واكد العضو السابق في المنتخب الوطني الیاباني لكرة القدم، تسویوشي كیتازاوا، على دور الریاضة في بناء جسور التواصل قائلا: " كل ما یجول بخاطرك حول الألعاب الریاضیة أصبح ممكنا لأن العالم أصبح یلعب كفریق واحد”.

It is worth mentioning that when the tool was asked to spot mistakes and explain the corrections it made, it did not identify all the errors from the first prompt and often lacked in-depth explanations. Moreover, at times, it hallucinated, providing incorrect or irrelevant details. Thus, when the tool is applied to medical, legal, financial, or technical texts, this adequate performance, even slight errors or ambiguity, would cause damage consequences. Therefore, while the tool is valuable, it requires care and validation in high-stakes contexts.

## Results and discussion

ChatGPT-4o's post-editing and human post-editing performance were evaluated by three human evaluators (EV1, EV2, EV3) across several linguistic aspects: Fluency, Accuracy, Efficiency, Terminology, Consistency, Cohesion, Syntax, Grammar, and Cultural for performing the quantitative and qualitative analysis. The results are measured on a 5-point Likert scale where 1 = Poor, 2 = Fair, 3 = Good, 4 = Very Good, and 5 = Excellent. After collecting the evaluators' rating scores, we applied a paired *t*-test for our statistical analysis because of its effectiveness in comparing differences between ChatGPT-4o and human post-editing and determining whether the observed differences were statistically significant, providing a reliable and quantitative assessment of the comparative performance, the average score for each aspect is depicted in Table 12.

**Table 12:** Human evaluator's scores for ChatGPT-4o and human post-editing performance across various.

**Evaluators**	**Post-editors**	**Fluency**	**Accuracy**	**Efficiency**	**Terminology**	**Consistency**	**Cohesion**	**Syntax**	**Grammar**	**Cultural appropriateness**
EV1	ChatGPT-4o	4	4	5	3	3	2	4	4	3
	Human	5	5	2	5	4	5	5	5	5
EV2	ChatGPT-4o	3	4	5	3	2	2	4	3	4
	Huma	5	5	2	5	5	5	5	5	5
EV3	ChatGPT-4o	4	3	5	3	3	3	4	3	3
	Huma	5	5	3	5	5	5	5	5	5

**Figure 1:**
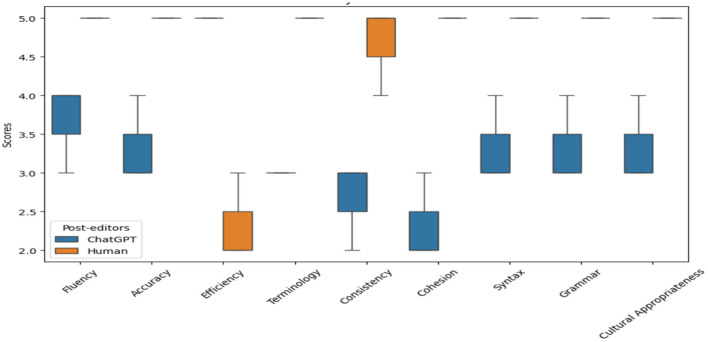
ChatGPT-4o and human post-editing across nine metrics. Human post-editing outperforms in all categories except efficiency, with higher medians and tighter interquartile ranges (IQRs) (orange boxes), indicating superior consistency in quality, accuracy, and fluency. ChatGPT-4o (blue boxes) shows lower ratings and wider IQRs, reflecting variability in handling nuanced language, terminology, and grammar. While ChatGPT-4o maintains fluency and coherence due to its conversational design, it struggles with technical terms and syntactic precision. Its strength lies in speed, making it useful for time-sensitive tasks. However, human expertise remains essential for high-quality translations requiring cultural and linguistic nuance.

The box-and-whisker plot in Figure 1 shows the average ratings for ChatGPT-4o and human post-editing across nine evaluation metrics, showing that human post-editing consistently outperforms ChatGPT-4o in terms of performance, with significantly higher ratings in all categories except efficiency. This highlights the superiority of human editors in maintaining quality, accuracy, cultural appropriateness, and fluency in translations, as seen from the higher median lines and smaller interquartile ranges (IQRs) in the orange boxes for human post-editing. The IQR indicates low variance and better overall performance. In contrast, ChatGPT-4o shows lower ratings across these aspects with larger IQRs in the blue boxes, suggesting more variability and lower overall performance than human performance. This reflects a common challenge in ChatGPT-4o's handling of nuanced and culturally specific elements and their idiomatic meaning. It shows some deficiency in language smoothness and syntax, such as agreement errors, word order, and grammatical mistakes related to articles used, as seen in the analysis section.

In addition, ChatGPT-4o shows significant issues in the use of correct and consistent terminological and technical terms and failed to effectively post-editing. It still appears fluent **(**Maintaining logical flow and coherence between sentences and paragraphs**)**, precise, consistent in style and tone, and readable throughout the content due to ChatGPT-4o's conversational nature. Indeed, ChatGPT-4o has the potential for rapid processing and editing, making it a valuable tool for scenarios where speed is critical. While ChatGPT-4o excels in speed and efficiency, human post-editing remains crucial for achieving high-quality translations across these critical aspects.

**Figure 2:**
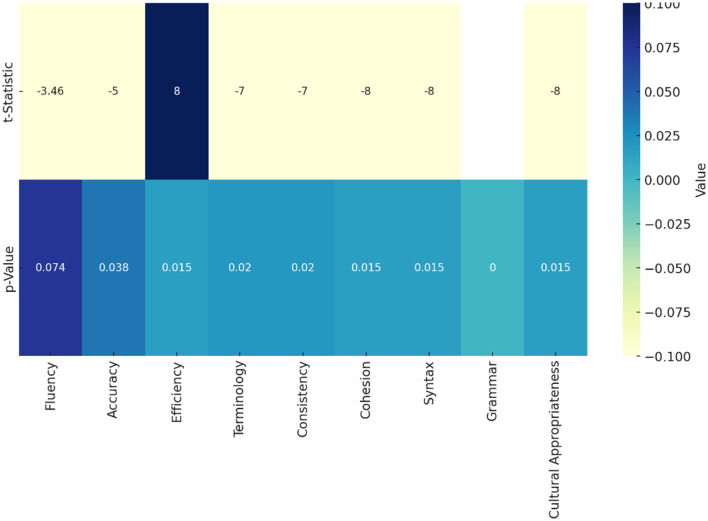
*T*-statistics and p-values comparing ChatGPT-4o and human post-editing. The *p*-value gradient (bottom half) shows statistical significance, with green (*p* < 0.05) indicating meaningful differences. Most aspects are green, except fluency (yellow). The top half shows *t*-statistics, revealing that human post-editing outperforms ChatGPT-4o in accuracy, terminology, consistency, cohesion, syntax, grammar, and cultural appropriateness (*t* = −3.46 to −8, *p* < 0.05). However, ChatGPT-4o excels in efficiency (*t* = 8.00, *p* = 0.015). Fluency shows no significant difference (*t* = −3.5, *p* = 0.074). The heatmap highlights ChatGPT-4o's efficiency advantage but confirms most aspects of human post-editing's superior quality.

The heat map in Figure 2 interprets the *t*-statistic and *p*-value values for each aspect when comparing ChatGPT-4o and human post-editing. The *p*-value gradient in the heatmap (represented in the bottom half of the heatmap) highlights statistical significance, with green indicating significant differences (*p* < 0.05). Most aspects are shaded green, confirming the reliability of the observed differences, except for fluency, which is shaded yellow. The *t*-statistic values are represented in the heatmap's top half, showing the direction and magnitude of differences in ratings. The *t*-statistics indicate that human post-editing generally outperforms ChatGPT-4o in most aspects, such as accuracy, terminology, consistency, cohesion, syntax, grammar, and cultural appropriateness, all showing significant negative values (ranging from −3.46 to −8) and corresponding *p*-values below 0.05, confirming that the differences are not only substantial but also statistically significant. However, regarding efficiency, ChatGPT-4o is rated significantly higher, with a positive *t*-statistic of 8.00 and a *p*-value of 0.015, indicating that it is more efficient than human post-editing. The only aspect where the difference is not statistically significant is fluency, with a *t*-statistic of −3.5 and a *p*-value of 0.074, suggesting that both methods perform similarly. Overall, the heatmap underscores ChatGPT-4o's strength in efficiency but highlights human post-editing's superiority in maintaining quality and accuracy across most aspects.

This study shows that, to some extent, ChatGPT-4o plays an influential role in improving the post-editing of machine-generated translations (MGT) in various domains attributed to its potential to generate fluent and natural translation reflecting relevant context and literature that is relatedly supporting the findings of Jiao et al. (2023) and Hendy et al. (2023). According to Peng et al. (2023), adapting ChatGPT-4o with optimized prompts and context improves its performance and makes it more suitable for specialized translation tasks. However, ChatGPT-4o's results may be similar to Google Translate or inaccurate without such optimization. Although ChatGPT-4o cannot provide completely accurate translations without human intervention, such integration would significantly reduce costs, time, and effort and provide considerable improvements and suggestions. Our analysis found that ChatGPT-4o can effectively contribute to post-edit generation and help identify translated content that may require further consideration or refinement. The results generated by ChatGPT-4o eliminate the need for skilled linguists to manually review the text, catch errors, give appropriate feedback, and ensure cultural appropriateness (Khan, 2024; Yang et al., 2023). To assess to which extent the three evaluators agree in their rating and thus ensure their reliability, we calculated the Inter-Annotator Agreement (IAA) using Spearman's rank correlation coefficient for pairwise comparisons and Fless' Kappa with quadratic weighted for overall agreement as illustrated in Table 13. The evaluators exhibit a near-perfect agreement for human post-editing, with pairwise Spearman's rho value of 0.99 and Fless'Kappa value of 0.85. For ChatGPT4o editing, the evaluators' agreement with pairwise Spearman's rho value is 0.85, and the Fless'Kappa value is 0.78, which means there is a substantial agreement among the three human evaluators.

**Table 13:** Inter-annotator agreement (IAA) scores.

**Metrics**	**ChatGPT-4o post-edits**	**Human post-edits**
Average pairwise Spearman's *rho*	0.85	0.99
Fless'Kappa (quadratic weights)	0.78	0.95

The values of IAA indicate a high level of reliability across the three evaluators (EV1, EV2, and EV3), stressing the robustness of our evaluation process of both human editors and ChatGPT4o as an editor.

## Conclusion

This research provides valuable insights into ChatGPT-4o's potential to enhance the MGT post-editing service and its overall role in assisting human translators with post-editing tasks in various domains. This study evaluates the post-editing performance of ChatGPT-4o compared to human editing based on an evaluation by three human raters on multiple metrics. The results show that although human post-editing outperforms ChatGPT-4o in most evaluation metrics, the latter provides a fluent translation, which promises to improve quality, work efficiency, and translation workflows in various fields. Additionally, the study found that ChatGPT-4o's detailed guidance includes clear task instructions, contextual information, and a description of the desired results that will help improve ChatGPT-4o's functionality. Future research may explore ChatGPT versions' use within professional translation services, especially in enhancing post-editing workflows, addressing the practical challenges, and identifying strategies to overcome these obstacles. Additionally, domain-specific fine-tuning of large-scale language models (LLMs) using specialized translation datasets needs exploration. Furthermore, creating and using diverse datasets that reflect a broader spectrum of Arabic dialects and text complexities to improve the generalizability and robustness of LLMs in translation tasks.

## Data Availability

The original contributions presented in the study are included in the article/supplementary material, further inquiries can be directed to the corresponding author/s.

## Footnotes

^1^ https://news.un.org

^2^ https://newatlas.com/robotics/robot-designed-to-perform-breast-examination

^3^ https://www.saudigazette.com.sa/article/609348

^4^ https://americanliterature.com/
